# May-Thurner Syndrome in a Case of Left Iliofemoral Vein Thrombosis With Contralateral Tubo-Ovarian Abscess in the Post-postpartum Period

**DOI:** 10.7759/cureus.49879

**Published:** 2023-12-03

**Authors:** Anupama Bahadur, Rajlaxmi Mundhra, Anoosha K Ravi, Poonam Gill, Anjali Pathak, Shreya Singhvi, Komal Shah, Gayatri Suresh

**Affiliations:** 1 Obstetrics and Gynecology, All India Institute of Medical Sciences, Rishikesh, Rishikesh, IND

**Keywords:** tubo-ovarian mass, thromboembolism, puerperium, deep vein thrombosis, ilio-femoral vein thrombosis

## Abstract

May-Thurner syndrome (MTS) is a rare, yet important, differential diagnosis in reproductive-age women with deep vein thrombosis (DVT). It is characterized by the compression of the left common iliac vein by the right common artery against the lumbar vertebra. The condition is complicated by recurrent DVT with pulmonary thromboembolism (PTE). Here is a case of multiparous women in early puerperium with right tubo-ovarian abscess and left lower limb DVT likely due to MTS. The diagnosis was further complicated by the presence of persistent thrombocytosis but a myeloproliferative neoplasm was ruled out by genetic mutation testing. She was given anticoagulants, and laparotomy was done for the excision of the tubo-ovarian mass in view of the persistent fever not responding to injectable antibiotics. PTE in the postoperative period was managed by anticoagulants followed by an inferior vena cava (IVC) filter for the risk of recurrent DVT and/or PTE in an MTS case.

## Introduction

May-Thurner syndrome (MTS) is a relatively uncommon cause of deep vein thrombosis (DVT), which accounts for 2-3% of all lower limb DVTs [1]. It is identified by the chronic compression of the left common iliac vein by the right common iliac artery against the lumbar vertebra. This compression of the left iliac vein brings about endothelial damage in the vessel and the consequent formation of a thrombus. A high index of suspicion is needed for the diagnosis of MTS, especially when there are other pre-existing hyper-coagulable risk factors like puerperium, pelvic mass, and thrombocytosis, like in the present case. This diagnosis is relevant because timely detection of such a condition enables one to undertake measures like an inferior vena cava (IVC) filter for the risk of pulmonary thromboembolism in the face of recurrent DVT.

## Case presentation

A 27-year-old multiparous woman who had an uneventful vaginal delivery 20 days ago came to our institution with a history of pain abdomen, distension abdomen, left lower limb swelling, and fever for two weeks. She had a history of laparotomy and left ovarian cystectomy for a possible benign cyst seven years ago. She had no co-morbidities, and there was nothing significant in her family history.

On examination, the general condition was fair. She had left lower limb edema with a cystic mass in the right hypochondrium, right lumbar, and right iliac regions with a smooth surface, restricted mobility from side to side, and no local rise of temperature.

**Figure 1:**
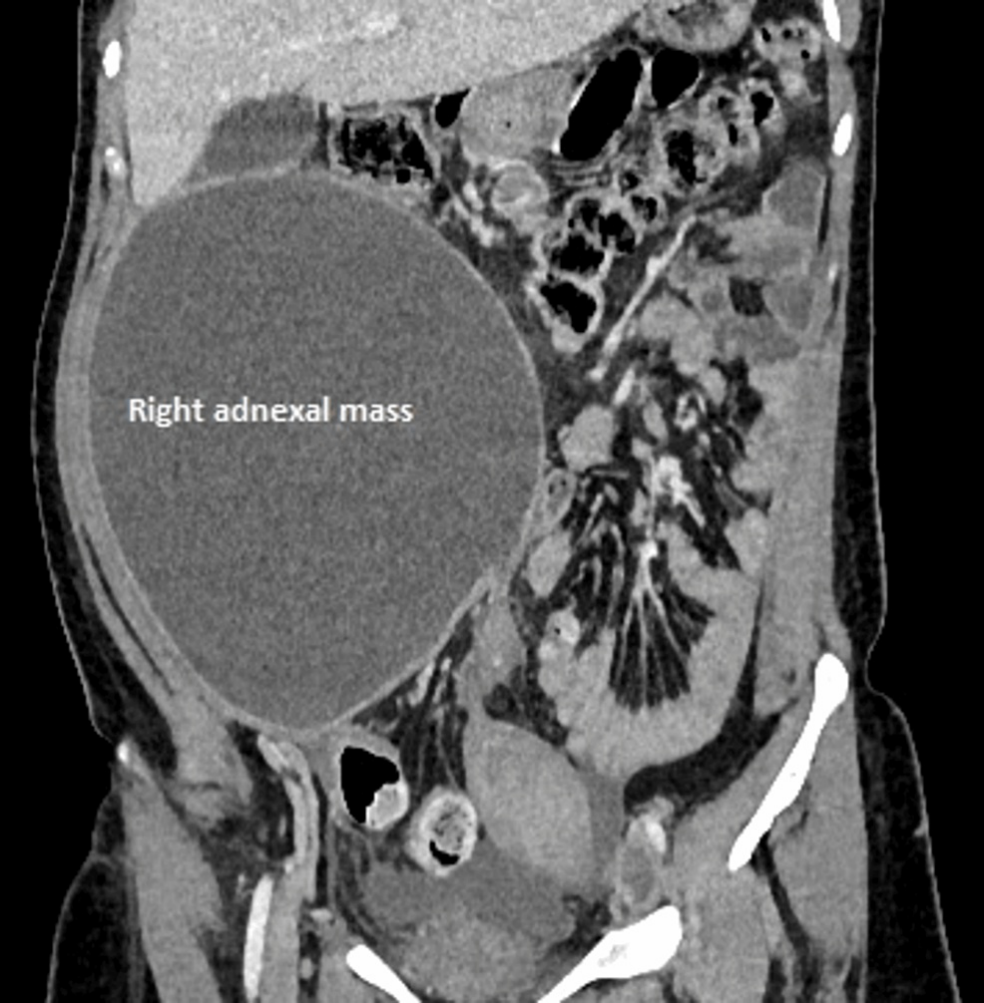
Contrast-enhanced computed tomography image Contrast-enhanced computed tomography of the abdomen and pelvis (coronal section) showing a right adnexal mass

Upon routine blood investigations, the patient was found to have thrombocytosis (6.6 L/mm^3^). All tumor markers were within normal limits. Also, deep venous thrombosis of the left common femoral vein (CFV), superficial femoral vein (SFV), and popliteal veins were seen on duplex ultrasound. Ultrasonography of the abdomen revealed a 15x17 cm solid cystic mass in the right adnexa with a moderate amount of ascites. With the suspicion of a neoplastic ovarian mass, contrast-enhanced computed tomography (CECT) of the abdomen and pelvis (Figure 1) was done, which revealed a right abdominopelvic mass of 11.6x16.2x20.6 cm arising from the right hemipelvis, seen extending into the right cornua of uterus likely fallopian tube/ovary. Mild ascites and bilateral pleural effusion were noted with iliac and paraaortic lymph nodes, the largest measuring 10 mm. The extent of the thrombus was the left common iliac, external iliac, and visualized superficial and deep femoral vein superiorly reaching upto IVC.

**Figure 2:**
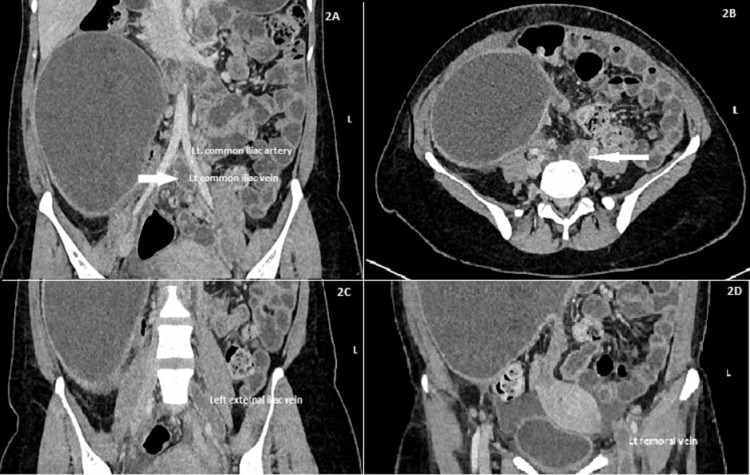
CT venography images CT venography (A) coronal section and (B) axial section showing left common iliac vein thrombosis (white arrow) and compression by left common iliac artery suggestive of May-Thurner syndrome; coronal sections showing the extent of thrombosis to (C) the left external iliac vein and (D) the left femoral vein.

To study the exact extension of DVT, computed tomography (CT) venography was done; thrombosis was found extending from the left common iliac vein to the proximal anterior and posterior tibial veins with compression of the left common iliac vein by the left common iliac artery, likely May-Thurner syndrome (Figures 2A-2D).

Although it was an atypical case, wherein the pelvic mass was originating from the right adnexa but venous thrombosis was in the left leg, the added high-risk factors like puerperal period and probable sepsis or neoplasm-induced thrombocytosis made it seem not so unlikely for such a presentation. When the CECT abdomen of the patient showed the proximal extent of thrombosis upto IVC, a hematologist's opinion was taken and CT venography was done. This imaging modality gave an uncommon differential diagnosis of May-Thurner syndrome. The patient was immediately started with unfractionated heparin infusion and the adequacy of anticoagulant therapy was monitored with aPTT. Eventually, it was changed over to low molecular weight heparin after a hematology consultation. Injectable antibiotics were given in view of febrile illness but only to find little improvement.

**Figure 3:**
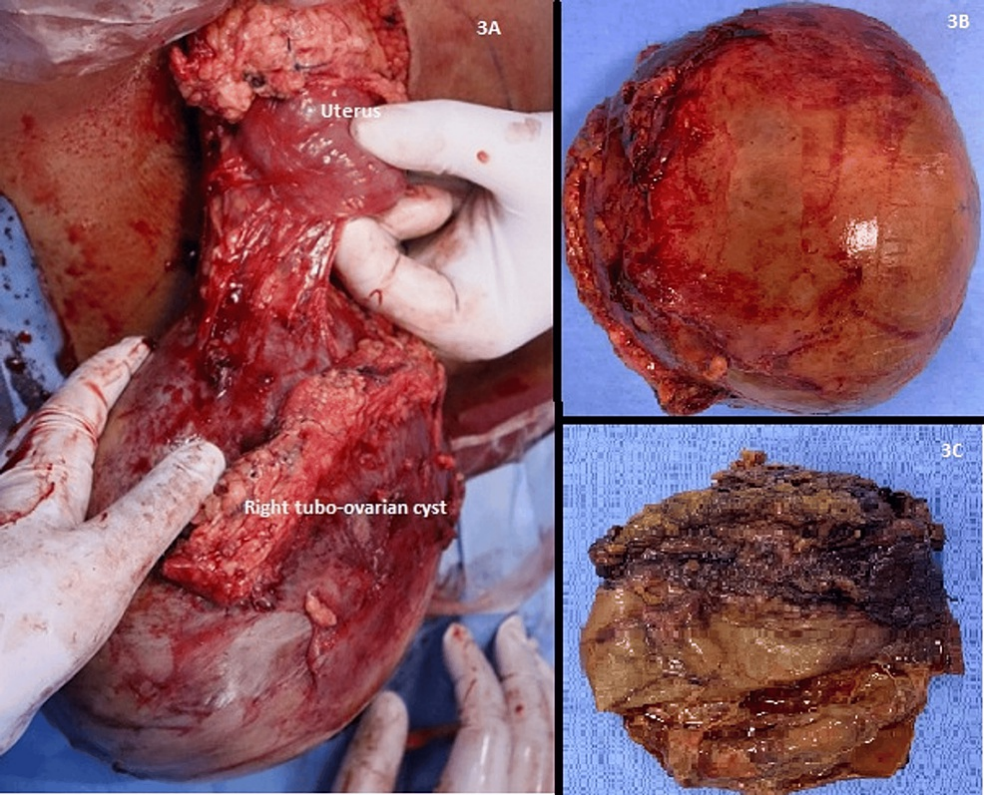
Intraoperative findings (A) Intraoperative findings suggestive of a right tubo-ovarian mass and a normal uterus. (B) Excised right tubo-ovarian abscess with (c) a cut section showing necrotic material within.

Given the persistent fever and the pelvic veins likely being compressed by the adnexal mass leading to thrombosis, the decision was taken to excise the mass. Laparotomy was done, and a tortioned right tubo-ovarian cyst of about 20×15 cm weighing approximately 1853 gm was found with areas of necrosis within (Figures 3A-3C). Uterus and left adnexa were normal looking with no obvious disease elsewhere in the abdomen or pelvis or retroperitoneal lymph nodes. Peritoneal washings were collected for cytology. Right salphingo-oophorectomy was done with an omental biopsy. The histopathological examination showed features of marked necrosis with chronic inflammation in the right ovarian cyst and no evidence of malignancy in peritoneal fluid or omentum.

Low molecular weight heparin was restarted within 24 hours of surgery. At 34 hours after surgery, the patient had sudden onset tachycardia. A CT pulmonary angiography was done and pulmonary thromboembolism (PTE) was detected in the left descending pulmonary artery and its branches. She was kept under intensive care, and an immediate bolus of unfractionated heparin 5000IU was given followed by continuous infusion with activated partial thromboplastin time (aPTT) monitoring. On postoperative day 5, oral anticoagulation with warfarin was added to low molecular weight heparin. International normalized ratio (INR) was monitored regularly and the dose of warfarin was titrated accordingly and low molecular weight heparin was withdrawn. In the postoperative period, fever subsided completely but thrombocytosis (8-8.6L/mm3) persisted. The patient was tested for a genetic mutation in JAK2V617F, BRC-ABL1, CALR, and MPL to rule out myeloproliferative neoplasm and was found negative. After a week of continuous anticoagulation, CT venography was repeated. Thrombus was found involving the left ascending and descending pulmonary artery and its branches and thrombosis of the left common iliac vein and popliteal vein persisted. An IVC filter was placed on postoperative Day 9 due to the possible risk of recurrent pulmonary thromboembolism and the patient was discharged on oral anticoagulants (warfarin). The patient has been taking regular oral anticoagulants for the past three months and shows no signs and symptoms of post-thrombotic syndrome. The stent was removed after three months and she is doing well post-surgery.

## Discussion

The occurrence of venous thromboembolism (VTE) is five times higher in pregnancy and the postpartum period, with an incidence of 1.72 to 2 per 1000 births [2], and it is an important cause of pregnancy-related morbidity and mortality. May-Thurner syndrome or iliac vein compression syndrome is a less recognized cause of iliofemoral DVT in women of reproductive age [3]. Anatomically, it is represented by the compression of the left common iliac vein by the overlying right common iliac artery against the lumbar spine [4]. This compression causes intraluminal collagen deposition in the vein and sluggish blood flow. If the venous obstruction is partial, the patient may remain asymptomatic or can develop symptoms associated with chronic venous hypertension such as lower extremity swelling and venous claudication. Rarely, other variations of MTS have been described, for instance, the compression of the right common iliac vein by the right common iliac artery or the compression of the left common iliac vein by the left common iliac artery as in the present case [5].

The diagnosis of MTS requires a high index of suspicion, particularly while encountering women during pregnancy or postpartum period, with acute unilateral left lower limb swelling [6]. The clinical features that should prompt one to aggressively pursue the diagnosis of MTS, are pain or swelling of the entire limb, dominance of venous claudication, no or inadequate relief of symptoms despite treatment of DVT, and unilaterality of symptoms, especially on the left side.

MTS generally requires a demonstration of the venous stenotic lesion in a suitable anatomic location. The most useful modality for the diagnosis of MTS is a venous duplex ultrasound, the sensitivity and specificity for which have been reported to be 91% and 99%, respectively [7,8]. Additional confirmation can be done by computed tomographic (CT) or magnetic resonance (MR) venography, both of which have a >95% sensitivity and specificity. The diagnosis of MTS in our patient was established by a CT venography, which revealed compression of the left common iliac vein by the left common iliac artery which is one of the variants of MTS.

In this case, the patient had a tubo-ovarian abscess, which was a likely cause of compression of pelvic veins. This along with the hyper-coagulable milieu created by pregnancy and sepsis, led to DVT in the first week postpartum. Though MTS was an added possibility, the tubo-ovarian abscess was still the first differential in the patient management owing to the less common presentation (compression of the left common iliac vein by the left common iliac artery) in an already rare diagnosis. The decision to excise the adnexal mass was also the need of the hour due to the persistent fever caused by the necrotic mass. An IVC filter could have been placed pre or intraoperatively to prevent the first episode of pulmonary thromboembolism (PTE). Nevertheless, the grave complication of PTE was managed effectively with timely anticoagulants under intensive care. The persistence of thrombosis despite adequate anticoagulation made the diagnosis of May-Thurner syndrome more of a reality. Thus, an IVC filter was placed to prevent further such episodes of PTE. The fact that MTS can predispose postpartum patients to proximal thrombi and subsequent PTE has been observed by other authors as well [9]. These can be treated successfully with thrombolysis and the risk of PTE can be minimized by the timely placement of an IVC filter. The case was further complicated by the persistence of thrombocytosis but a myeloproliferative neoplasm was ruled out by testing for genetic mutation in JAK2V617F, BRC-ABL1, CALR, and MPL.

## Conclusions

May-Thurner syndrome is characterized by unilateral left lower limb DVT due to the compression of the left common iliac vein by the right common iliac artery. Variants of MTS include compression of the left common iliac vein by the left common iliac artery. A high index of suspicion is needed for the diagnosis of MTS, especially when there are other pre-existing hyper-coagulable risk factors like puerperium, pelvic mass, and thrombocytosis. Persistence of thrombus or no relief in symptoms despite adequate anticoagulation should prompt the possibility of MTS. MTS can be managed by thrombolysis and the risk of PTE can be minimized by the timely placement of an IVC filter.
